# Too Old for Surgery? A Case Report of an Octogenarian Patient With Locally Advanced Lung Cancer Achieving Long‐Term Survival via Bilobectomy Followed by Adjuvant Targeted Therapy

**DOI:** 10.1002/cnr2.70652

**Published:** 2026-08-10

**Authors:** Han‐Yu Deng, Jia‐Hao Li, Xiao‐Jun Tang, Qinghua Zhou

**Affiliations:** ^1^ Department of Thoracic Surgery West China Hospital, Sichuan University Chengdu China; ^2^ Lung Cancer Center West China Hospital, Sichuan University Chengdu China; ^3^ West China School of Medicine Sichuan University Chengdu China

**Keywords:** adjuvant targeted therapy, lung cancer surgery, non‐small‐cell lung cancer, octogenarian

## Abstract

**Background:**

An increasing number of octogenarian patients are being diagnosed with lung cancer, but they are less likely to receive surgery because of fragility. As a result, the optimal management of lung cancer in octogenarians remains unclear. Here we report a case of an octogenarian patient with locally advanced non‐small‐cell lung cancer (NSCLC) who achieved long‐term survival via surgery followed by adjuvant targeted therapy.

**Case:**

An 82‐year‐old male patient was admitted to our center for lung cancer surgery in April 2020 at West China Hospital, Sichuan University. He was clinically diagnosed with cT2N1M0(cIIB, AJCC 8th edition) lung cancer in the right lower lobe. Because of his good cardiopulmonary function without comorbidities, the patient was scheduled for thoracoscopic surgery. However, due to tumor invasion into the middle lobe and dense adhesions to the bronchus and vessels, the patient underwent conversion to open thoracotomy and finally received bilobectomy with systematic lymph node dissection. The patient recovered uneventfully and was discharged on postoperative day 5. After surgery, the patient was pathologically diagnosed with pT3N2M0(pIIIB) lung adenocarcinoma harboring an EGFR L858R gene mutation and began adjuvant icotinib. The patient progressed with multiple pulmonary metastasis at 13 months after starting icotinib and his liquid biopsy revealed a T790M mutation; he began treatment with the third‐generation EGFR‐TKI aumolertinib. In the last follow‐up in December 2025, the patient remained on aumolertinib therapy and remained alive without progression.

**Conclusion:**

Age alone is not a contraindication for lung cancer surgery and octogenarian patients may tolerate surgical resection following careful preoperative evaluation. Adjuvant targeted therapy may prolong survival in octogenarian lung cancer patients harboring oncogenic driving gene mutation after surgery.

Lung cancer has become the leading cause of cancer‐related death among all malignancies worldwide [1], and in China, it also remains the most common malignant tumor with the highest mortality, imposing a great burden on the healthcare system [2]. Even though lung cancer patients are often diagnosed between 63 and 77 years old [3], octogenarian (80 years of age or older) patients represented the most rapidly growing group, accounting for about 14% of all lung cancer patients; they were also less likely to be subjected to radical therapy such as surgery, yielding inferior outcomes compared with younger patients [4]. Therefore, the optimal therapeutic strategies are urgently needed for improving the prognosis of octogenarian lung cancer patients. A previous study confirmed that even with increased age, surgery is feasible and safe with good long‐term outcomes after careful preoperative evaluation for elderly lung cancer patients [5]. However, as for adjuvant therapy, although previous trials have confirmed adjuvant epidermal growth factor receptor (EGFR) tyrosine kinase inhibitors (TKIs) may improve the prognosis of NSCLC patients with EGFR mutation after surgery [6, 7], the feasibility and effects of adjuvant EGFR‐TKIs for octogenarian NSCLC patients remain unclear because of a lack of available evidence especially focusing on surgery and adjuvant targeted therapy in octogenarians. Therefore, in this study, we reported an octogenarian patient with locally advanced NSCLC who was successfully managed with surgery followed by adjuvant EGFR‐TKIs and achieved long‐term survival. Written informed consent was obtained from the patient for the publication of case details and use of images. To our knowledge, this is the first case report focusing on patients over 80 years old who achieved long‐term survival via lung cancer surgery and adjuvant EGFR‐TKIs.

## 1 Case Report

**FIGURE 1:**
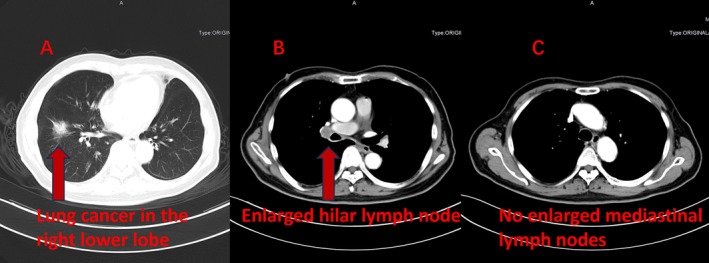
Preoperative computed tomography images of the octogenarian patient. (A) Lung cancer in the right lower lobe. (B) Enlarged lymph node in the hilum. (C) No enlarged lymph nodes in the mediastinum.

**FIGURE 2:**
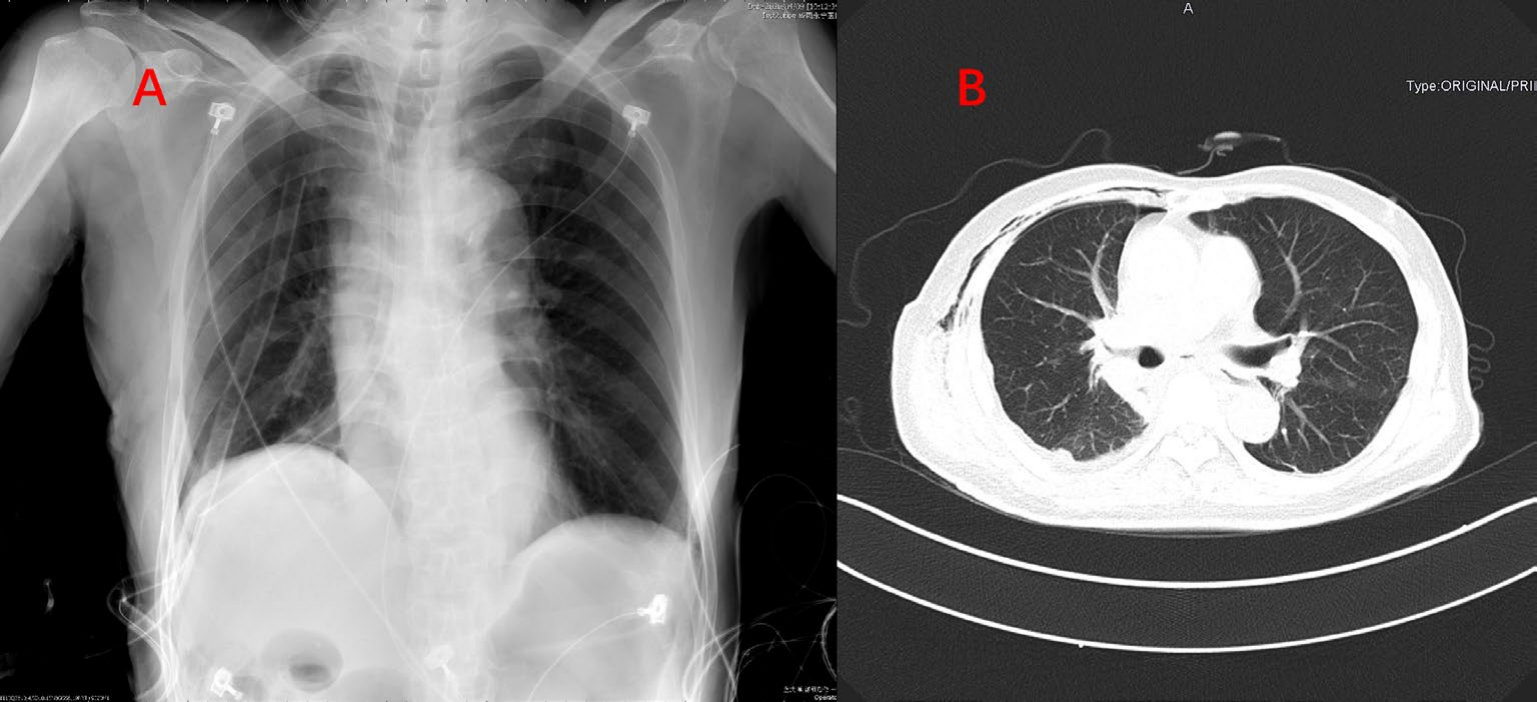
Postoperative X‐ray and computed tomography images of the octogenarian patient. (A) X‐ray image. (B) Computed tomography image.

An 82‐year‐old male patient was admitted to our center in April 2020 complaining of cough for over 6 months and hemoptysis for 1 week at West China Hospital, Sichuan University. His computed tomography (CT) showed a lung nodule approximately 2.1 × 2.2 cm in size in the anterior basal segment of the right lower lobe, suspected to be lung cancer (Figure 1). Only interlobar lymph nodes between the middle and lower lung bronchus were found to be enlarged, and no enlargement of mediastinal lymph nodes was noticed with negative findings on positron emission tomography (PET)/CT scan. As a result, invasive mediastinal lymph node staging was not performed. Percutaneous transthoracic needle biopsy confirmed the diagnosis of lung adenocarcinoma. His brain magnetic resonance image revealed no brain metastasis and PET/CT revealed no apparent abnormal positive findings in extra‐thoracic organs. Moreover, the patient had no co‐morbidities such as hypertension, diabetes, or coronary heart disease and no history of smoking or alcohol consumption. His cardiopulmonary function tests showed that ejection fraction (EF) value was 65% and forced expiratory volume in 1 s (FEV1) was 2.5 L. As for preoperative laboratory tests, the level of hemoglobin was 168 g/L and the platelet count was 212 × 10^9^/L. His preoperative prothrombin time was 10.3 s with normal liver function (alanine aminotransferase: 14 IU/L, aspartate aminotransferase: 17 IU/L) and renal function (serum creatinine: 88 μmol/L). However, the level of his cytokeratin‐19 fragment (CYFRA21‐1) was 5.02 ng/mL, with normal level of carcinoembryonic antigen (2.06 ng/mL). Therefore, after careful discussion in our multidisciplinary team (MDT), the patient was clinically staged as cT2N1M0 (Stage IIB) according to the 8th edition of TNM stage system [8], and based on the guidelines [9], the patient was advised to undergo video‐assisted thoracoscopic lobectomy and systematic lymph node dissection. During surgery, the enlarged interlobar lymph nodes and the primary tumor were found to have invaded the right middle lung lobe and severe density and adhesion of the hilar were encountered; as a result, the patient underwent conversion to open thoracotomy and finally received bilobectomy of the right middle and lower lobes with systematic lymph node dissection. Fortunately, the patient recovered uneventfully and was discharged on postoperative day 5 (Figure 2). As for pathological diagnosis, the lesions were located in the right lower lobe invading the right middle lobe of the lung, and histological diagnosis was invasive adenocarcinoma with papillary predominant subtype (approximately 2% micro‐papillary subtype) and poor differentiation. Positive pleural involvement and spread through air spaces were noticed in the tumor. Lymph node evaluation showed tumor metastasis in both mediastinal and intrapulmonary lymph nodes. His immunohistochemistry results were as follow: CK7(+), TTF‐1(+), NapsinA(+), PSA(−), elastic fibers(+), ALK‐V(−), and ROS‐1(−). The patient was pathologically staged as pT3N2M0 (Stage IIIB) and next‐generation sequencing (NGS) test of the tumor tissue revealed an EGFR L858R mutation and a *TP53* G245D mutation.

**FIGURE 3:**
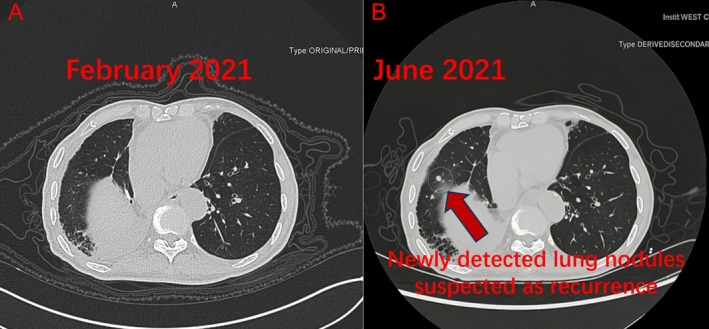
Computed tomography images of the octogenarian patient showing newly detected lung nodules in the right lung lobe suspected to be recurrent lesions. (A) No sign of recurrence in February 2021. (B) Newly detected lung nodules suspected as recurrence.

**FIGURE 4:**
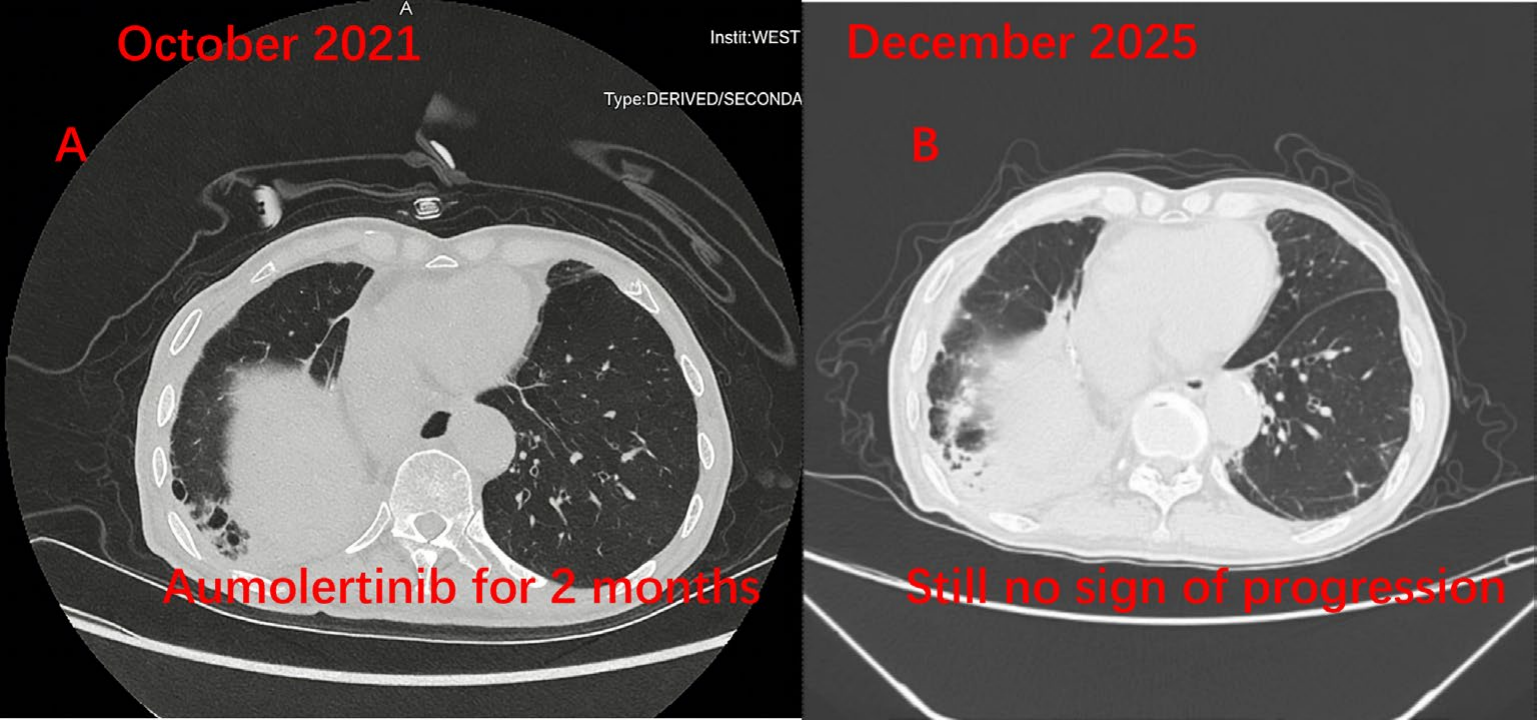
Follow‐up computed tomography images of the octogenarian patient after receiving aumolertinib as second‐line treatment. (A) Computed tomography after taking aumolertinib for 2 months. (B) Computed tomography in December 2025 showing no sign of recurrence.

**FIGURE 5:**
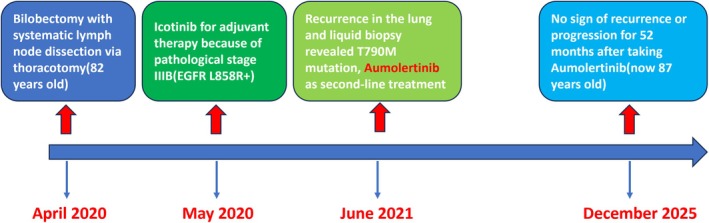
Treatment timeline of the octogenarian patient.

After surgery, the patient was advised to undergo adjuvant therapy and he refused adjuvant chemotherapy and agreed only to receive adjuvant targeted therapy because he was afraid of the side effects of chemotherapy as well as decreased physical tolerance after major surgery. In 2020, in China, the first‐generation EGFR‐TKI, icotinib, was approved for adjuvant targeted therapy for EGFR‐positive NSCLC after surgery because the EVIDENCE trial met its endpoints and yielded positive results [6]. Therefore, the patient started adjuvant icotinib therapy from May 2020 without any adverse effects and was regularly followed up every 3 months. However, in June 2021, the patient was found to have multiple lung nodules in the right remaining lung lobe (Figure 3) with an elevated tumor biomarker CYFRA 21‐1 (5.28 ng/mL, normal range: < 3 ng/mL) after receiving the first‐generation EGFR‐TKI for 13 months. Moreover, the newly detected lung nodules increased continuously after 2‐month observation and were suspected to be tumor recurrence after MDT discussion. Because the largest newly detected lung nodule was only 0.9 cm in diameter, re‐biopsy of the nodule for pathological diagnosis and tissue NGS was not feasible. Because previous studies have identified the utility of liquid biopsy in identifying EGFR driver and acquired resistance mutations with good sensitivity for various blood‐based biomarkers [10], liquid biopsy served as an alternative to figure out the mechanisms for therapy decision making, especially when re‐biopsied tumor tissues were not available. Therefore, the patient underwent liquid biopsy for NGS test which is regularly carried out in West China Hospital, Sichuan University; the liquid biopsy revealed not only the EGFR L858R mutation but also the T790M mutation, which is the most common gene mutation conferring acquired resistance to the first‐generation EGFR‐TKIs. As a result, the patient started third‐generation EGFR‐TKI therapy from August 2021. In China, aumolertinib was approved for the second‐line therapy after resistance to the first‐generation EGFR‐TKIs harboring the T790M mutation on the basis of the APOLLO trial [11]. After taking aumolertinib for about 3 months, the newly detected lung nodules in the remaining right lung lobe disappeared and the tumor biomarker CYFRA 21‐1 became normal (2.28 ng/mL), which confirmed the efficacy of second‐line aumolertinib (Figure 4). The patient continued the second‐line aumolertinib treatment without any adverse effects and was still regularly followed up every 3 months for the first 2 years and every 6 months thereafter; in our last follow‐up in December 2025, the patient remained alive (now he is over 87 years old) and his latest CT scan still showed no sign of progression (Figure 4). Here we have summarized the treatment timeline of the patient in Figure 5; the patient has remained progression‐free for 52 months after second‐line aumolertinib treatment.

## 2 Discussion

In this study, we reported a case of an octogenarian NSCLC patient who successfully underwent bilobectomy with systematic lymph node dissection. After surgery, the patient received adjuvant targeted therapy and achieved long‐term survival (more than 5 years). To our knowledge, this is the first case of an extremely elderly patient achieving long‐term survival after lung cancer surgery followed by adjuvant EGFR‐TKIs.

As the population aging progresses, the diagnosis and treatment of elderly patients with NSCLC have become a key focus in clinical medicine. Previously, older patients were often advised to undergo conservative therapy due to declining physiological functions, multiple comorbidities, and increased surgical risks [12]. However, recent studies have indicated that age is not the primary barrier to surgical intervention [13]. For elderly patients with stable physical conditions and no severe co‐morbidities (such as severe coronary artery stenosis, severe chronic obstructive pulmonary disease, and so forth), aggressive radical surgery can significantly improve survival rates [14]. In our previous study, we also found that surgery especially lobectomy might improve the prognosis of octogenarian NSCLC patients after careful preoperative evaluation [15]. Therefore, relying on age alone as the criterion for surgical contraindications is inadequate. For elderly patients with NSCLC, a comprehensive evaluation of physiological status, cardiopulmonary function, comorbidities, and tumor stage is essential for informed decision‐making. When these conditions permit radical resection, surgical approach remains a preferred option, significantly improving long‐term survival rates and quality of life. In our case, the patient had good cardiopulmonary function and had no co‐morbidities, and surgery was indicated for him. Even though, the patient underwent bilobectomy via open thoracotomy, he recovered uneventfully. Therefore, octogenarian patients can tolerate surgical resection after careful evaluation, especially in the era of minimally invasive thoracic surgery. Moreover, conversion from minimally invasive surgery to open thoracotomy was also feasible for these patients. Therefore, careful preoperative evaluation seemed more important for octogenarian NSCLC patients considered for surgery. Currently, the potential value of artificial intelligence in the preoperative evaluation of octogenarian patients is being extensively investigated [16], and novel prediction models are badly needed for decision‐making regarding surgery for octogenarian NSCLC patients.

Postoperative adjuvant therapy is a crucial component of comprehensive treatment for patients undergoing surgery for resectable NSCLC. It may eliminate residual microscopic lesions after surgery, reduce the risk of local recurrence, and significantly improve long‐term survival [17]. For NSCLC patients with EGFR mutations, adjuvant targeted therapy and chemotherapy have become standardized treatment approaches, and numerous clinical studies have demonstrated that this regimen may offer notable advantages in delaying disease progression [18]. Although first‐generation EGFR‐TKIs failed to show an overall survival benefit for resectable NSCLC harboring EGFR mutation in the ADJUVANT trial [19] and in the IMPACT trial [20], the EVIDENCE trial comparing icotinib and chemotherapy in the adjuvant setting for Stage II–IIIA NSCLC harboring EGFR mutation found that compared with chemotherapy, icotinib significantly improved survival with a better tolerability profile in patients with EGFR‐mutant Stage II–IIIA NSCLC after surgery [6], leading to the approval of icotinib as adjuvant targeted therapy for EGFR positive NSCLC in China. Therefore, in our case, the patient received icotinib as adjuvant therapy and tolerated it well. However, the patient developed metastasis at 13 months after starting icotinib, which was in line with previously reported progression‐free survival time of icotinib. As we all know, drug resistance inevitably occurs with first‐generation EGFR‐TKIs. The mechanisms for acquired drug resistance included target gene modification (such as T790M mutation), alternative pathway activation (such as mesenchymal‐epithelial transition amplification) and histological or phenotypic transformation (such as transformation to small‐cell lung cancer) [21]. In our patient, his liquid biopsy showed a T790M mutation which was one of the important mechanisms of acquired drug resistance to the first‐generation EGFR‐TKIs and started to receive aumolertinib for second line treatment. Aumolertinib, a novel third‐generation EGFR inhibitor independently developed in China, demonstrates better selectivity for sensitive mutations and the ability to overcome T790M resistance compared with first‐ and second‐generation inhibitors, while also exhibiting good safety and efficacy profiles [22]. In the APOLLO trial [11], aumolertinib was confirmed to be an effective and well‐tolerated third‐generation EGFR‐TKI for patients with EGFR T790M‐positive advanced NSCLC after disease progression on first‐ and second‐generation EGFR‐TKI therapy, which was approved in China for patients positive for EGFR T790M NSCLC. In this case, the patient demonstrated a remarkable efficacy and safety profile with aumolertinib in a second‐line treatment regimen. The patient's chest CT imaging confirmed complete remission of multiple nodular lesions in the right lung after 2 months of aumolertinib therapy. As of December 2025, the last follow‐up, the patient remained alive without recurrence or metastasis and had no adverse effects, validating the effectiveness of aumolertinib in treating EGFR T790M NSCLC.

Recently, in the ADAURA study, the third‐generation EGFR‐TKI, osimertinib demonstrated more significant recurrence risk reduction in postoperative adjuvant therapy and exhibited excellent metastasis prevention capabilities in the central nervous system (CNS) with improved CNS disease‐free survival (DFS) leading to its approved for adjuvant therapy for resectable NSCLC harboring EGFR mutation [7]. Furthermore, osimertinib provided a significant overall survival benefit as adjuvant therapy [23]. Moreover, in the ARTS trial [24], aumolertinib was also found to yield a statistically significant and clinically meaningful improvement in DFS in patients with Stage II–IIIB NSCLC with EGFR mutation after surgery as adjuvant therapy, which underscores the promise of aumolertinib in fulfilling an unmet need for effective adjuvant EGFR‐targeted therapy in resectable NSCLC. Therefore, with more EGFR‐TKIs being approved for adjuvant targeted therapy, the prognosis of locally advanced NSCLC harboring EGFR mutation may be further improved. However, there are no available guidelines for the management of postoperative recurrence in octogenarian NSCLC patients after surgery. A previous study found that after carefully selecting patients for intensive therapy, such as those with a good performance status, antitumor treatment may lead to longer survival after postoperative recurrence in octogenarians [25]. Therefore, it seems important to comprehensively evaluate the physical status and gene mutations of octogenarians who developed postoperative recurrence. Moreover, our case also highlighted the importance of liquid biopsy technology in investigating resistance mechanisms in NSCLC. Previous studies have identified the utility of liquid biopsy in the identification of EGFR driver and acquired resistance with good sensitivity for various blood‐based biomarkers [10], liquid biopsy served as an alternative to figure out the mechanisms for therapy decision making, especially when re‐biopsied tumor tissues are not available. With its minimally invasive, efficient, and repeatable nature, this technique enables real‐time monitoring of tumor genomic dynamics, facilitating the development of personalized precision treatment plans [26]. However, further research is needed to better increase the validity and efficacy of liquid biopsy in the management of solid tumors.

Even though we reported the first case of an octogenarian NSCLC who achieved long‐term survival with surgical resection followed by adjuvant EGFR‐TKIs, our study still has several limitations. First, we only drew our conclusion base on a case report, which could impact the validity of our conclusions. Second, even though our case recovered uneventfully after conversion to thoracotomy with bilobectomy, it is important to carefully perform preoperative risk evaluation and more efforts are needed to better select octogenarians for lung cancer surgery. Third, our case only received the first‐generation EGFR‐TKIs for adjuvant targeted therapy, and whether receiving the third‐generation EGFR‐TKIs as up‐front adjuvant therapy could improve the prognosis of the patient remained unclear. Finally, our case underwent liquid biopsy for detection of drug resistance and received the second‐line aumolertinib treatment. As is well known, drug resistance still exists and how to deal with recurrence or progression in the future remained to be resolved. Therefore, our conclusions should be taken with caution and further similar cases are encouraged to add evidence to our conclusions. Despite the above limitations, our case report served as a valuable preliminary observation that warrants further investigation in larger cohorts.

## 3 Conclusions

Age alone is not a contraindication for lung cancer surgery and it is safe and feasible to perform lung cancer surgery for octogenarians provided careful preoperative evaluation is conducted. Adjuvant targeted therapy may improve the prognosis of octogenarian NSCLC patients after surgery with oncogenic driver gene mutation safely and effectively; in the case of recurrence or progression, figuring out the mechanisms of drug resistance and choosing corresponding therapy may further improve long‐term survival.

## Author Contributions

**Han‐Yu Deng:** writing – original draft, formal analysis. **Jia‐Hao Li:** formal analysis, investigation. **Xiao‐Jun Tang:** conceptualization, writing – review and editing. **Qinghua Zhou:** conceptualization, writing – review and editing, supervision.

## Funding

This work was supported by the Department of Science and Technology of Sichuan Province (2022JDKP0009).

## Ethics Statement

This work was approved by the Ethics Committee of West China Hospital of Sichuan University (No. 2026‐207). The patient provided his written informed consent to participate in this study.

## Conflicts of Interest

The authors declare no conflicts of interest.

## Data Availability

The datasets supporting the conclusions of this article are included within the article and its additional files. All methods were performed in accordance with the relevant guidelines and regulations. The dataset for this article is publicly available, available upon reasonable request, or data sharing.
